# Assessing Adherence in the CAPRISA 004 Tenofovir Gel HIV Prevention Trial: Results of a Nested Case–Control Study

**DOI:** 10.1007/s10461-014-0753-8

**Published:** 2014-03-20

**Authors:** Kathleen M. MacQueen, Mark A. Weaver, Francois van Loggerenberg, Stacey Succop, Nelisle Majola, Doug Taylor, Quarraisha Abdool Karim, Salim Abdool Karim

**Affiliations:** 1 FHI 360, 359 Blackwell Street, Suite 200, Durham, NC 27701 USA; 2 The University of North Carolina at Chapel Hill, Chapel Hill, NC USA; 3 CAPRISA, University of KwaZulu-Natal, Durban, South Africa

**Keywords:** Microbicides, Tenofovir gel, Adherence, HIV prevention, Self-report

## Abstract

Adherence undeniably impacts product effectiveness in microbicide trials, but the connection has proven challenging to quantify using routinely collected behavioral data. We explored this relationship using a nested case–control study in the CAPRISA 004 Tenofovir (TFV) gel HIV prevention trial. Detailed 3-month recall data on sex events, condom and gel use were collected from 72 incident cases and 205 uninfected controls. We then assessed how the relationship between self-reported adherence and HIV acquisition differed between the TFV and placebo gel groups, an interaction effect that should exist if effectiveness increases with adherence. The CAPRISA 004 trial determined that randomization to TFV gel was associated with a significant reduction in risk of HIV acquisition. In our nested case–control study, however, we did not observe a meaningful decrease in the relative odds of infection—TFV versus placebo—as self-reported adherence increased. To the contrary, exploratory sub-group analysis of the case–control data identified greater evidence for a protective effect of TFV gel among participants reporting less than 80 % adherence to the protocol-defined regimen (odds ratio (OR) 0.30; 95 % CI 0.11–0.78) than among those reporting ≥80 % adherence (Odds Ratio 0.81; 95 % CI 0.34–1.92). The small number of cases may have inhibited our ability to detect the hypothesized interaction between adherence and effectiveness. Nonetheless, our results re-emphasize the challenges faced by investigators when adherence may be miss-measured, miss-reported, or confounded with the risk of HIV.

## Introduction

Women constitute 57 % of the population living with HIV in sub-Saharan Africa, with few options to negotiate and enact behaviors to reduce their risk [1]. Results from a vaginal microbicide trial demonstrated proof of concept that a gel containing an antiretroviral confers partial protection against HIV acquisition. CAPRISA 004 was a double-blind, randomized controlled trial comparing pericoital use of 1 % tenofovir (TFV) gel with placebo gel in 889 sexually active women aged 18–40 years in urban and rural KwaZulu-Natal, South Africa [2]. The success of the trial represents a major breakthrough for addressing women’s biological vulnerability to the virus, but it is tempered by the moderate estimated 39 % protective effect in the intention-to-treat analysis [95 % confidence interval (CI) 6–60 %]. The lack of effectiveness observed in a more recent study of daily use of 1 % TFV gel [3] also raises concerns regarding the willingness of women to sufficiently adhere to microbicide use.

In the CAPRISA 004 study, participants were asked to vaginally apply a first dose of the assigned study product within 12 h before coitus and apply a second dose as soon as possible but within 12 h after coitus; they were also advised to only use two doses within any 24-h period. At each monthly visit participants were asked how many times they had sex in the previous 30 days. They were also asked to return unused and empty (i.e., presumably used) applicators; applicators that were not returned were assumed to be unused. In the primary manuscript [2], gel adherence was calculated as the estimated proportion of reported sex acts covered by two doses, calculated for each woman by dividing half the number of returned empty applicators by the number of reported sex acts that month. Using this composite measure the median adherence level was estimated to be 60.1 %, and the estimated effectiveness of TFV gel was higher among women with greater than 80 % adherence (54 vs. 39 % overall).

The coitally-related gel-use message created challenges for measuring adherence and evaluating the potential contribution of behavioral variability to the effectiveness outcome. However, the costs of collecting more detailed recall data on sex acts and gel use from all CAPRISA 004 trial participants was prohibitive. In addition, participants may have been unlikely to provide unbiased detailed recall data on a monthly basis, given the other requirements of trial participation. We therefore chose to conduct a nested case–control study in real time within the trial, an approach that had been independently recommended as a means of linking adherence patterns to HIV incidence in an Institute of Medicine Report on methodological challenges in prevention trials [4]. Our primary objectives were to statistically model the odds of HIV infection for women in the TFV gel group compared to those in the placebo group while controlling for reported gel use, and to qualitatively evaluate patterns of gel use behavior among participants. Here we report the statistical modeling results.

## Methods

CAPRISA 004 participants were recruited from a rural and an urban site in KwaZulu-Natal, South Africa. The rural site was situated in the KwaZulu-Natal midlands, about 150 km north-west of Durban, and housed at the CAPRISA Vulindlela Research Clinic, adjacent to a comprehensive primary health care clinic. The urban site was located at the CAPRISA eThekwini Research clinic, adjacent to a sexually transmitted infections clinic in the Durban city center. The trial began enrollment in May 2007, and recruitment for the nested case–control study began 10 months later. At each monthly visit, CAPRISA 004 participants were tested for HIV with two rapid HIV tests. Participants with either positive or discordant results were identified as potential cases in the nested case–control study; those who were never confirmed positive by PCR-RNA were subsequently excluded. Unmatched controls were recruited using the following procedures. For each month of the trial, five target dates were randomly selected for each site from among those dates when the clinics were scheduled to see participants. At least five CAPRISA 004 participant identification numbers were randomly drawn from the list of scheduled participants to accommodate the possibility of participants refusing enrolment or missing their appointment. Women previously interviewed were excluded from the control participant recruitment list. Additional inclusion criteria for the case–control study included: enrolled in the CAPRISA 004 trial for at least 2 months; interviewed within 6 weeks from the date of the rapid test when recruitment was initiated; and gel use not suspended (e.g., due to pregnancy) for the entirety of the behavioural recall period.

The Time-Line Follow-Back (TLFB) method was used to collect detailed 3-month recall data on sexual events and gel use. Originally developed to assess alcohol use, the TLFB has previously been applied in HIV behavioral research settings [5]. The TLFB combines findings from cognitive psychology about the value of memory aids to facilitate recall with open-ended interviewing techniques to facilitate collection of detailed behavior patterns over extended time intervals. The structural features of the TLFB have been shown to facilitate recall of sexual behaviors occurring up to 90 days earlier [5]; this timeframe encompasses the period during which HIV infection most likely occurred among cases in our study. We incorporated a number of strategies into the interview to improve recall, including use of “special days” that are generally meaningful (e.g., holidays, pay days, travel away from home), menstrual cycles, “anchor days” defined by the participant rather than the interviewer, and visual aids modified from some developed as part of counseling for the CAPRISA 004 adherence support program [6]. Quantitative data were double-entered and all interviews were audio recorded using digital recorders. The TLFB data were recorded on calendar forms during the interview using standardized notation and then transferred to data entry forms. As needed, the interview audiotape was reviewed to facilitate completion of the data entry forms. At random intervals throughout the study, a sample of recordings was independently reviewed and compared with transcripts and data collection forms by the CAPRISA study coordinator, a native Zulu speaker.

Two gel use exposure variables were derived for the case–control study: the proportion of vaginal sex acts covered by a double dose of gel and the proportion covered by at least a single dose within 12 h of coitus. To determine whether a participant adhered to the protocol-defined double-dose regimen for any particular vaginal sex act, it was necessary to determine whether any gel was used within 12 h prior to the act and within 12 h following the act. If complete dates and times were provided for all relevant vaginal sex acts and gel uses, then the calculation could be done directly. However, if the participant did not know the exact event time, data collectors used a 24-h “clock-face” to help participants estimate a time range for each vaginal sex act and gel insertion. The clock-face was originally developed for the CAPRISA 004 trial as a tool to help women determine the timing of the two gel insertions and was therefore a familiar way for study participants to describe the timing of events [6]. Culturally meaningful time ranges were defined at varying levels of specificity and participants were then asked to recall the time of the event with as much specificity as possible. For example, an event that took place after sunrise and before sunset could be coded as “Daytime, unknown time” or more specifically as “Afternoon, unknown time” (12h00–18h00) or more specifically yet as “Afternoon, early” (12h00–14h00).

For each vaginal sex act or gel use for which a time code was used instead of exact time, minimum and maximum possible date/times were derived as described above. All possible differences between the minimum and maximum times of the sex act and gel use were then calculated. If any gel use could have occurred within 12 h prior to a sex act, then the act was classified as being covered by a pre-act gel use. Similarly, if any gel use could have occurred within 12 h following a sex act, then the act was classified as being covered by a post-act gel use. Each gel use was classified as either a pre-act use or a post-act use, but not both, with pre-act taking precedence unless there was a previous gel use that could also be classified as pre-act. This derivation thus gave the participant the benefit of the doubt with regard to correct timing of gel use.

Several steps were taken to reduce the potential for biased elicitation on the part of the case–control study interviewers due to knowledge of a participant’s HIV infection status. The interviewers were not told which days were randomly selected for recruitment of control participants, in order to prevent the interviewer from determining whether a particular participant was recruited as a case or a control. The case–control study coordinators informed the case–control interviewers who should be recruited and when, but did not provide any information on HIV test results. The case–control study interviewers did not have access to any CAPRISA 004 trial participant files or attend CAPRISA 004 trial staff meetings where participant details were discussed. Despite these efforts, it was not completely possible to blind interviewers if the participant was interviewed after HIV post-test counseling and chose to reveal her test results.

The potential for differential recall bias according to whether or not the participant knew her HIV status at the time of interview was another concern. At the urban site, participants continued with other study procedures while awaiting results of their HIV test, and every effort was made to interview case–control study participants before they received their results from the post-test counselor. At the rural clinic, participants observed the result of the rapid test with the counselor. Consequently, all case–control participants at the rural site were interviewed by the TLFB method after receiving their HIV test results. In view of this potential for differential recall bias, we included knowledge of HIV status at time of interview in covariate-adjusted analyses described below.

## Data Analysis

*T* tests for continuous variables and Chi-squared tests for categorical variables were used to compare cases and controls on baseline and 3-month recall variables. Separately for the double-dose and any gel use exposure variables, logistic regression models were fit that included effects of treatment group assignment, adherence as a continuous variable (0–100 % in the 3-month recall period), and the interaction between treatment group and adherence. Estimated odds ratios (ORs) for the model parameters were computed along with 95 % CIs and p values. Adjusted models included effects of age, time in study, site (urban, rural), total number of vaginal sex acts in the recall period, the proportion of acts where a condom was used, and knowledge of infection status when interview took place.

Our primary interest was in the treatment by adherence interaction due to the potential for the relationship between adherence and effectiveness to be confounded by unmeasured HIV risk factors. For example, if high adherers were less likely to have infected partners (an unobserved factor) then even an ineffective product could appear to reduce the risk of HIV acquisition. The interaction should be less impacted by this type of confounding so long as the decision to use gel was not impacted by treatment group assignment (as expected when using a blinded placebo comparator). Analogously, we could expect the OR for infection (TFV gel vs. placebo) to be lower in a subgroup of high adherers than in a subgroup of low adherers if effectiveness increases with adherence and if risk factors are balanced between groups. The interaction term in our model was used to assess this relationship over a continuous, rather than categorical, adherence measure. However, we also explored the effectiveness by adherence subgroups, with high adherence defined as ≥80 %.

## Results

We enrolled 72 of the 98 (73 %) confirmed HIV-infected CAPRISA 004 participants in the nested case–control study (Table 1); of the 26 infected women who were not enrolled, seven became infected before the case–control study initiated and 19 could not be interviewed within 6 weeks of their positive rapid test. All 205 of the CAPRISA 004 participants recruited as controls consented to participate, and six later became cases.

**Table 1:** Enrollment in case–control study, by site

Study arm	Durban (urban)		KwaZulu-Natal (rural)		Total
	Cases	Controls	Cases	Controls	
1 % TFV gel	11	60	15	50	136
Placebo	18	48	28	47	141
Total	29	108	43	97	277

*TFV* tenofovir

In descriptive analyses, cases were on average younger than controls at the time of enrollment in the CAPRISA 004 trial (22.7 and 24.2 years respectively; p = 0.03) and their sexual partners were on average younger than those of controls (26.0 and 27.9 years respectively, p = 0 .02; Table 2). Cases and controls were similar with regard to the number of months enrolled in the trial, but differed with regard to timing of the interview (before or after HIV post-test counseling) and the average number of days between HIV testing and the interview (Table 3).

**Table 2:** Baseline characteristics among cases and controls

	Cases (n = 72)	Controls (n = 205)	p value
1 % Tenofovir gel arm	36.1 %	53.7 %	.01
Mean age (in years)	22.7	24.2	.03
Monthly income <R1000	76.3 %	83.4 %	.37
Married	2.7 %	6.3 %	.25
Stable partner	94.4 %	89.2 %	.20
Mean age at sexual debut	17.1	17.4	.30
Mean number sexual partners (in lifetime)	2.9	3.0	.88
Mean age of oldest partner (past 30 days)	26.0	27.9	.02
Reported sex in the past 7 days	54.1 %	62.4 %	.22
Always use condom during sex	33.3 %	32.6 %	.92
Reported new partner (past 30 days)	0.0 %	1.4 %	.30
Reported anal sex (past 30 days)	0.0 %	0.4 %	.55

**Table 3:** Timing of interview among cases and controls

	Cases (n = 72)	Controls (n = 205)	p value
Months enrolled in CAPRISA 004 trial: mean	10.4	10.9	0.52
Interviewed after HIV post-test counseling:* n* (%)	62 (86.1)	109 (53.2)	<0.01
If yes, days between HIV test date and interview date: mean	16.2	2.9	<0.01

The number of vaginal sex acts reported during the 3-month recall period varied somewhat for cases (mean = 14.1) and controls (mean = 16.9, p = 0.07; Table 4), and the mean proportion of vaginal sex acts covered by condom use was lower among cases (0.68) than controls (0.80; p = 0.02). The mean percentage of vaginal events covered by a double dose of gel was smaller for cases (0.69) than controls (0.75), but this difference, unadjusted for treatment group, was not significant (p = 0.10). The corresponding mean percentage of acts covered by at least one gel was smaller for cases (0.79) than controls (0.86; p = 0 .03).

**Table 4:** Sex, gel use, and condom use reported by cases and controls in 3-month recall period

All participants	Cases	Controls^a^	p value
	n = 72	N = 201	
Mean number of vaginal sex events	14.1	16.9	0.07
Mean percent of acts with condom used	0.68	0.80	0.02
Mean percent of acts with double dose of gel	0.69	0.75	0.10
Mean percent of acts with at least one gel use	0.79	0.86	0.03

1 % TFV arm	n = 26	N = 108	
Mean number of vaginal sex events	14.0	17.3	0.22
Mean percent of acts with condom used	0.65	0.80	0.06
Mean percent of acts with double dose of gel	0.74	0.75	0.82
Mean percent of acts with at least one gel use	0.80	0.87	0.15

Placebo arm	n = 46	N = 93	
Mean number of vaginal sex events	14.2	16.5	0.19
Mean percent of acts with condom used	0.71	0.80	0.16
Mean percent of acts with double dose of gel	0.67	0.75	0.07
Mean percent of acts with at least one gel use	0.79	0.85	0.17

*TFV* tenofovir

^a^Two placebo and two 1 % TFV gel controls reported no sex acts during recall period

We observed a significant overall association of TFV gel group assignment on the odds of HIV infection (adjusted OR 0.49, 95 % CI 0.26–0.90, p = 0.02), consistent with the fact that cases were selected from a trial population with fewer infections in the TFV group. Increasing rates of self-reported adherence were associated with somewhat lower odds of infection in both the placebo and TFV gel groups (results not shown), although these reductions were not significant for either the double-dose or any gel use variables. We observed a non-significant 6 % increase in the relative odds of infection (TFV vs. placebo) for each 10 % increase in adherence to the double-dose regimen (p = 0.61 for test of interaction) and a non-significant 3 % decrease in the relative odds of infection for each 10 % increase in the rate of any gel use (p = 0.78 for test of interaction).

The unexpected increasing OR for infection as self-reported adherence to the double-dose regimen increased triggered additional exploratory analysis of our data. Among participants reporting greater than 80 % adherence to the double-dose regimen (mean of 92 and 94 % adherence, respectively, for cases and controls), the odds of infection was not significantly different between the TFV and placebo groups (adjusted OR 0.81; 95 % CI 0.34–1.92; p = 0.63) (Table 5). However, there was a significantly lower odds of infection for the TFV group among participants reporting less than 80 % adherence (mean of 51 and 54 % adherence, respectively, for cases and controls) to the double-dose regimen (adjusted OR 0.30; 95 % CI 0.11–0.78; p = 0.01). The difference in ORs between subgroups was not significant, however, in adjusted analysis (p = 0.14).

**Table 5:** Odds ratios for infection, overall and by exploratory sub-groups of self-reported adherence

	TFV		Placebo		Unadjusted		Adjusted^a^	
	Cases	Controls	Cases	Controls	OR (95 % CI)	p value	OR (95 % CI)	p value
Double-dose of gel								
<80 % Adherence	10	51	30	42	0.27 (0.12, 0.62)	<0.01	0.30 (0.11, 0.78)	0.01
≥80 % Adherence	16	57	16	51	0.89 (0.41, 1.97)	0.78	0.81 (0.34, 1.92)	0.63
Test of difference in ORs across subgroups						0.04		0.14
Any gel use								
<80 % adherence	8	25	17	22	0.41 (0.15, 1.14)	0.09	0.36 (0.10, 1.28)	0.11
≥80 % adherence	18	83	29	71	0.53 (0.27, 1.04)	0.06	0.54 (0.26, 1.10)	0.09
Test of difference in ORs across subgroups						0.69		0.70
Overall	26	108	46	93	0.49 (0.28, 0.85)	0.01	0.49 (0.26, 0.90)	0.02

*TFV* tenofovir

^a^Adjusted for location, time since enrollment, age, reported number of vaginal acts in 3-month recall period, knowledge of HIV status, and reported proportion of acts with condom use

## Discussion

Analysis of adherence previously reported for the CAPRISA 004 trial centered on a composite measure based on the reported number of sex acts and returned empty applicators at each monthly visit [2]. Although the corresponding test of interaction between adherence level and effectiveness was not reported, the results of sub-group analysis were consistent with TFV gel being more effective among women with higher adherence (54, 38 and 28 % reductions in risk for women with greater than 80, 50–80 and less than 50 adherence, respectively). While insightful, the composite measure does not distinguish between single and double gel use for any particular sex act.

The ability to tease out finer levels of variability in adherence through self-reported data would be a significant contribution, and we implemented a nested case–control study within the CAPRISA 004 trial to try and capture these effects. However, we did not identify meaningful associations between self-reported gel use patterns and risk of HIV. To the contrary, in exploratory analyses we found less evidence for the effectiveness of TFV gel among women reporting high (>80 %) adherence to the double dose regimen than among those reporting lesser adherence. It is possible that the relatively small number of cases (N = 72) provided insufficient power to detect meaningful interactions between gel use patterns and HIV infection, and our results could have been impacted by unmeasured confounding. It is also possible that a large percentage of participants who reported high adherence in the case–control study were substantially over-reporting that behavior, a result observed in other HIV prevention trials where non-adherence was verified using drug concentration data [7, 8]. Determining whether or not this occurred in our case–control study is challenged by infrequent collection of drug concentration specimens and the delay between the timing of sex and clinic visits when specimens were obtained. If such systematic report bias was occurring, however, it would have undermined our ability to identify relationships between adherence and effectiveness.

The potential impact of self-report bias was an important concern in the design of the case–control study, and steps were taken to both decrease and evaluate its impact. Women in the CAPRISA 004 trial were counseled monthly on correct use of the gel, and this may have induced women to over-report gel use. To reduce the potential for this kind of desirability response in the case–control study, participants were told that the CAPRISA 004 trial counselors and nurses would not have access to the case–control study files and the interviewers would not have access to the CAPRISA 004 trial files. Nonetheless, the average double dose adherence rate in the case–control study, 74 %, was very similar to the 72 % obtained from 30-day recall of total number of sex events and empty applicator returns in the trial. We further attempted to minimize recall bias through the use of memory aids and detailed probing during the collection of the calendar data. However, delays in the timing of the interview may have differentially affected recall accuracy in cases [9].

Operationally, the implementation of a nested case control study in real time with the CAPRISA 004 trial was successful and resulted in minimal disruption of clinical trial procedures. Trial participants were generally willing to partake in the one-time in-depth interview, although recruitment of seroconverting women was understandably more difficult and interviews were significantly delayed if recruitment took place after HIV post-test counseling. In addition to the need to be sensitive to the emotional state of the women, interviews were often deferred due to the many high-priority clinical procedures the women were asked to participate in such as confirming HIV infection, assessing viral load, and evaluating the potential for the emergence of TFV-resistant virus.

We demonstrated that nested observational studies allow for the collection of detailed behavioral data in real time with large scale HIV prevention trials. However, our results suggest that such studies may be challenged in their ability to support modeling of product effectiveness when sex acts leading to HIV infection may have occurred long before the interview, and without complementary knowledge regarding the potential confounding factor of HIV exposure among controls. Studies in discordant couples with more regular (e.g. daily or bi-weekly) product use may be a more viable setting for modeling the relationship between adherence and effectiveness with the nested case–control design. But even there, some validation of self-reported adherence data using objective drug concentration measures would be essential.
